# An evaluation of the impact of anti-rabies programs in Nigeria

**DOI:** 10.1097/MS9.0000000000000250

**Published:** 2023-02-17

**Authors:** Ridwan O. Adesola, Hafeez T. Akinniyi, Don E. Lucero-Prisno

**Affiliations:** a Department of Veterinary Medicine, Faculty of Veterinary Medicine, University of Ibadan, Ibadan, Nigeria; b Department of Global Health and Development, London School of Hygiene and Tropical Medicine, London, UK; c Faculty of Management and Development Studies, University of the Philippines Open University, Los Baños, Laguna, Philippines; d Faculty of Public Health, Mahidol University, Bangkok, Thailand

**Keywords:** Africa, anti-rabies, Nigeria, rabies

## Abstract

Rabies remains a significant public health problem in Africa with outbreaks reported in most countries. In Nigeria, the most populous country in Africa, rabies causes a significant public health burden partly due to uncoordinated efforts and the ineffectiveness of available anti-rabies programs. We aim to address the current efforts, and challenges associated with the available anti-rabies programs in Nigeria and provide recommendations to tackle these challenges.

**Results:**

The anti-rabies programs available in Nigeria are highlighted. They are sponsored by different bodies such as government parastatals, veterinary teaching hospitals, professional associations, nongovernmental associations, and students. These programs provide support to eradicate rabies but are not devoid of challenges. Recommendations are provided to the Nigerian government, bodies anchoring the anti-rabies programs, and health professionals to tackle the challenges hindering the effective impact of the programs.

**Conclusion:**

Anti-rabies programs in Nigeria are supported by individual and collaborative bodies. It is pertinent to hold on to these programs and create a comprehensive national program to achieve effective rabies eradication in Nigeria.

Highlights

- Rabies remains a public health importance issue.
- The number of anti-rabies programs available in Nigeria.
- Rabies is a big problem that needs a comprehensive national program.

## Introduction

Each year, rabies results in 10 million human exposures and thousands of fatalities. All warm-blooded animals are susceptible to rabies, which is primarily brought on by the rabies virus, a member of the *Lyssavirus* genus, in the family *Rhadoviridae* 1. With over 95% of rabies-related illnesses and mortality linked to interactions with domestic dogs, Africa and Asia have the highest burden^2^,^3^. According to information provided at the WHO expert consultative meeting in 2018, rabies spread by dogs accounts for 21,476 annual human fatalities in Africa and 55,000 in Nigeria^4^. Between 1978 and 2020, more investigations on the prevalence of dog rabies were conducted in the northern part of Nigeria. The prevalence of rabies virus antigen detection ranged from 3% to 28%^5^. According to studies on dog bites, most attacks were unprovoked (36.4–97%) and involved canines with low vaccination rates (12–38%)^5^.

The bulk of fatalities on the continent occurred in remote locations and among people who did not have access to health care, with mortality rates among small children being especially high^3^,^4^. To halt the epidemics of neglected tropical diseases, the third Sustainable Development Goal of the year 2030 called for improvements in health^3^. The World Organization for Animal Health, the WHO, and the Food and Agriculture Organization of the United Nations joined forces to support endemic nations in their efforts to eradicate canine-mediated rabies by 2030^6^.

**Figure 1:**
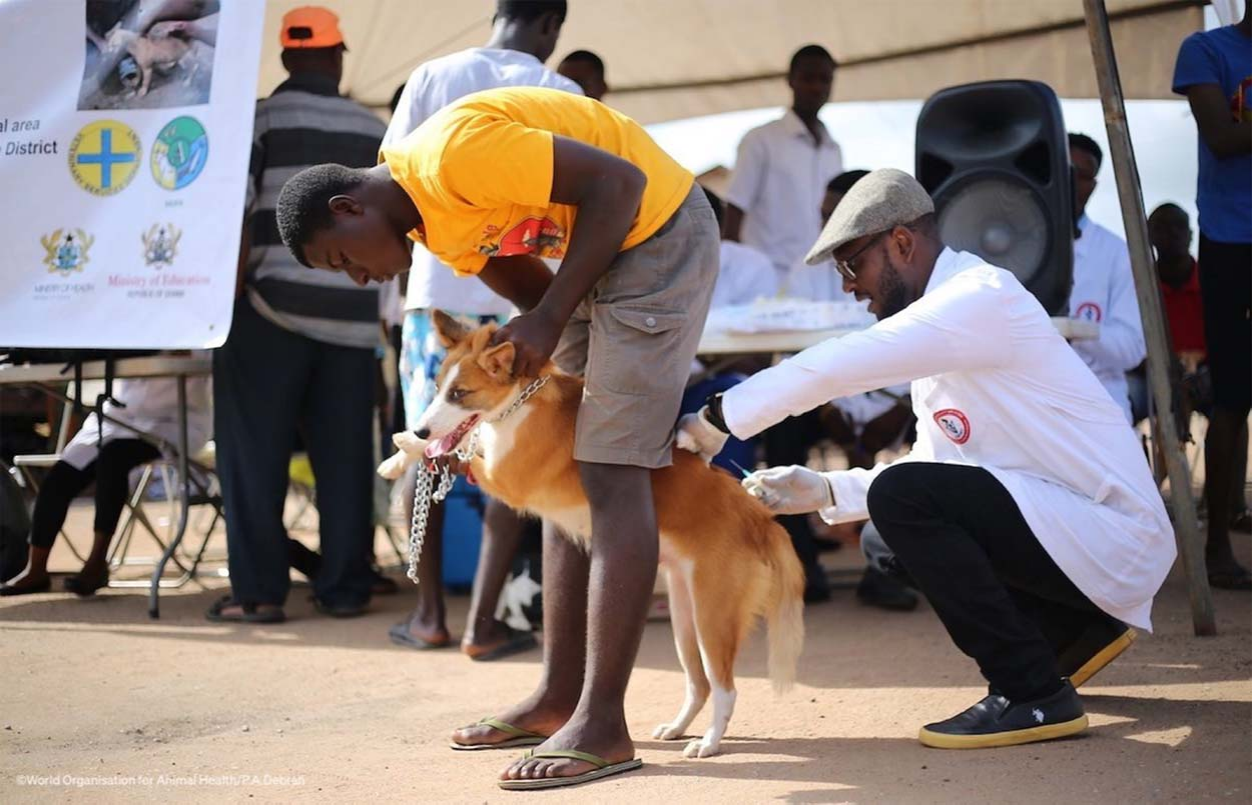
Diagram illustrating the vaccination process for rabies^8^.

In Nigeria, where canine rabies is not well-controlled and is most frequently transmitted by the bite of an infected dog, rabies continues to be a serious public health concern. Dogs have been implicated as the reservoir and transmitter of rabies in Nigeria in published papers and surveys^7^. Through the widespread administration of effective dog rabies vaccines (Fig. 1) and enforcement of responsible dog ownership, canine rabies can be eradicated. However, a sizable number of canines in Nigeria are unvaccinated, which has allowed the rabies cycle to continue to spread there^9^. Anti-rabies campaigns in Nigeria are usually organized annually by small individual bodies. Although a national rabies control program is nonexistent in the country, private and public institutions, professional bodies, student associations, and some not-for-profit organizations often organize sensitization and vaccination programs either individually or collaboratively. Veterinarians and veterinary organizations appear to take the forefront in this campaign. Owing to the lack of a comprehensive national program, the interventions are often limited to specific regions in the country. Therefore, this article aims to address the current efforts and challenges associated with the available anti-rabies programs in Nigeria and provide recommendations on how to improve these programs to achieve the 2030 zero rabies goal in Nigeria.

### Anti-rabies programs in Nigeria

#### Government parastatal

Through the Ministry of Agriculture, some state governments demonstrate exemplary leadership by executing awareness campaigns and free vaccination of dogs across their states. In 2021, the Lagos State Ministry of Agriculture, and Department of Veterinary Services, organized a mass anti-rabies vaccination campaign where 6250 vaccines donated by the World Health Organization for Animal Health through the Federal Ministry of Agriculture and Rural Development were administered to dogs and cats presented at the State Government Veterinary Clinics. The exercise also covered public sensitization, including school awareness campaigns, and members of the community were taught simple protocols to follow when bitten by a dog^10^.

#### Veterinary Teaching Hospitals (VTHs)

The 13 veterinary schools across the six geo-political zones of Nigeria have hospital facilities for training clinical veterinary students, while simultaneously catering to the demand for veterinary care in their respective communities. These VTHs also make significant contributions to the fight against rabies infection. The Veterinary Teaching Hospital, University of Jos, notably organizes anti-rabies campaigns. In 2018, the highlights of the University of Jos campaign are: sensitization visits to schools, rabies awareness walk, and subsidized vaccination of pets at the VTH^11^. The more recent edition of the campaign, organized by the institution in 2021, involved free vaccination of all companion pets above the ages of 3 months and public awareness of the dangers of the disease^11^. In March 2022, the Veterinary Teaching Hospital, University of Ibadan, conducted a 2-day anti-rabies vaccination campaign involving free vaccination of dogs at centers strategically situated within the University community.

#### Role of professional associations

The Nigerian Veterinary Medical Association (NVMA) is a national body of veterinarians comprising chapters across the 36 states of the federation and the Federal Capital Territory. Many of the state chapters rise to organize anti-rabies campaigns of varying magnitude. The Enugu State Chapter of the NVMA marked the 28th September 2017 by organizing a rabies awareness roadshow, hosting a radio discussion on rabies control and prevention on Dream FM, Enugu, and administering free anti-rabies vaccines at the State Veterinary Clinic, Uwani, Enugu, the Veterinary Teaching Hospital, University of Nigeria, Nsukka and the Veterinary Clinic, Government House, Enugu^10^.

In 2020, the Ogun State Chapter of the NVMA organized a free anti-rabies vaccination campaign and activities such as a rabies awareness walk, symposium, and press conference^8^. Also, the Kwara State Chapter of the NVMA marked the same event by conducting a state-wide anti-rabies campaign which was flagged off by the Deputy Governor of the State and targeted at vaccinating 5000 dogs in the rural areas within the state^10^.

Furthermore, 28 September 2021 was another opportunity ceased by some NVMA state chapters across the country for the annual campaigns. As done the previous year, NVMA Ogun State Chapter organized a seminar to educate the public on rabies, its prevention, and necessary first-aid measures after a dog bite episode. This was followed up with 1-week free anti-rabies vaccination of dogs at designated Veterinary hospitals within the State^10^. In the South-East, NVMA Enugu State Chapter also underscored the significance of the day with a special radio program on rabies prevention and control, a mega sensitization rally, and free anti-rabies vaccination outreach to selected rural areas under Enugu-East, Enugu-South, and Enugu-North Local Government Area. Anti-rabies vaccination was also extended to Enugu urban, with free vaccines administered to pets at every government veterinary clinic in the state and the Veterinary Teaching Hospital, University of Nigeria, Nsukka^10^. In the North-West, NVMA Kebbi State Chapter commemorated the day with a media outreach through local radio and television stations, a road rally, and free mass vaccination at three different locations within Kebbi state^8^. In the North-East, NVMA Taraba State Chapter also followed suit, sensitizing the public on the menace of rabies and offering free rabies vaccines to dogs and cats^10^.

#### Role of student associations

The onus of anti-rabies advocacy rests largely on veterinarians. However, students have also contributed significantly to the campaign against the disease, usually done in September. In 2019, One Health Unibadan, a students’ group at the University of Ibadan, visited some secondary schools in the University community to interactively engage and educate students on rabies infection^10^. Similarly, the International Veterinary Students’ Association, the University of Ibadan Chapter, marked the 28th of September 2019 by sensitizing secondary school students about the importance of vaccination in rabies prevention and elimination^10^.

In 2020, IVSA Ibadan continued with the visitation of over 10 schools and the enlightenment of about 500 secondary school students on rabies, and dog bite prevention^10^. Also in 2020, Animal Home, an association of students in Ladoke Akintola University of Technology, Ogbomoso, visited some rural communities in her environs to vaccinate dogs for free and sensitize community members on rabies and its prevention.

#### Private institutions

Private establishments, especially those that render veterinary services, also play their part in the fight against rabies. Some private veterinary practices and clinics have independently organized awareness campaigns every September, with vaccination of pets at capacities within their means. In commemoration of the 2019 World Rabies Day, Vetlane Animal Healthcare, ElSalem Estate, Lugbe, Abuja, conducted free anti-rabies vaccination of dogs and cats in Abuja Metropolis^10^. Also, Carotid Animal Health Services, Egbeda, Lagos, is another private veterinary organization that has shown consistency in the anti-rabies campaign in recent years. In 2020, the organization conducted a 7-day free anti-rabies vaccination of pets in the Egbeda community between the 20th and 27th of September, together with sensitization of the public on rabies infection in dogs and humans. A repeat of the campaign was organized to mark the 2021 World Rabies Day, following the template of the previous year^10^.

#### Nonprofit organizations

The contribution of not-for-profit organizations in the campaign against rabies infection has also been observed. In 2017, War Against Rabies Foundation, Ahmadu Bello University, Zaria, Kaduna, organized a weekly sensitization program featuring children rabies awareness campaigns in schools, rabies pamphlet distribution, and public information street walk. Also in 2017, Noah Bankole Initiative, Asokoro, Abuja, sponsored a short talk on African Independent Television (AIT) to sensitize the public on the causes, symptoms, diagnosis, and prevention of rabies^10^.

Animal Hospital Unit of onaaraTODAYservices, a non-governmental organization, organized a free vaccination campaign for dogs in Akanran village, Ibadan. This was done on 28 September 2018, with a system whereby well-to-do clients were convinced to pay for one more anti-rabies vaccination of another dog. The funds generated were then used to finance the free vaccination campaign in the village^10^.

In 2019, PawsConnect, a nonprofit organization in Garki, Abuja, conducted an awareness dog walk that featured a paper presentation on the significance of rabies vaccination and free vaccination of dogs^10^. In 2020, Isaachrist Awareness Foundation organized a door-to-door, village-to-village rabies awareness program in Abuja. The campaign was aimed at creating on-the-spot advisory support, media campaigns, roadshows, and a personal true-life e-book of a first-hand caregiver to a rabid patient.

#### Collaboration

Aside from the isolated efforts of individual groups and organizations to combat the menace of rabies in their respective communities, there have been occasions where some of these bodies joined forces to produce a greater impact. In 2018, the NVMA, Oyo State Chapter, collaborated with Family Medicine Department, University College Hospital, Ibadan, and the International Veterinary Students’ Association (IVSA), Ibadan, to organize a symposium titled: ‘The role of One Health initiative to achieving rabies eradication in Nigeria, by the year 2030.’ Professionals in the discipline of human and veterinary medicine were invited to educate the audience on rabies infection in humans and animals and its eradication using a One Health approach^10^.

As part of a community development project under the National Youth Service Corps scheme, some corps members conducted a seminar in 2019. This was done in collaboration with the NVMA, Ondo State Chapter. The seminar aimed at bringing animal and human health experts, legal experts, and the media under one banner to discuss the importance of One Health approach in rabies elimination in Nigeria by 2030^10^.

In 2020, the Department of Veterinary Services, Benue State Ministry of Agriculture partnered with the Benue State chapter of the NVMA and non-governmental organizations such as Tesedona Foundation for Animal Health, MoboVet, War Against Rabies Foundation, Zoetis, VetConnect, Life Stock Management Services, and Vaccinate 500 Project. The collaboration aimed to advocate for policymakers the need for requisite policies and legislation toward the successful elimination of rabies by 2030. It also featured a radio talk show and a 2-week free anti-rabies vaccination of dogs and cats in the Makurdi metropolis^10^ (Table 1).

**Table 1:** Anti-rabies programs between 2017 and 2021 in Nigeria^10^.

Organizer	Category	Program	Date	Objectives
War Against Rabies Foundation, Ahmadu Bello University, Zaria, Kaduna	Nonprofit organization	Children/public enlightenment	28 September–3 October 2017	Children's rabies awareness campaigns in schools and rabies pamphlet distribution, and public information street walks
Noah Bankole Initiative, Asokoro, Abuja	Nonprofit organization	Let’s talk about rabies	28 September 2017	A short talk on rabies, causes, symptoms, diagnosis, prevention and control on local television; African Independent Television (AIT) kakaaki
Veterinary Teaching Hospital, University of Jos	Veterinary Teaching Hospital	University of Jos World Rabies Day Annual Program	24–28 September 2018	Sensitization visit to schools, rabies awareness walk, partnership to enhance rabies prevention and control, subsidized vaccination at the veterinary teaching hospital
Nigerian Veterinary Medical Association (NVMA), Enugu	Professional body	World Rabies Day 2018 Enugu	27 September 2018	Rabies Awareness Road show, free anti-rabies vaccination, radio discussion on rabies control and prevention
Nigerian Veterinary Medical Association (NVMA), Oyo State Chapter, Family Medicine Department, University College Hospital (UCH), Ibadan, and the International Veterinary Students’ Association (IVSA), Ibadan	Collaboration	Symposium: The role of One Health initiative to achieving rabies eradication in Nigeria, by the year 2030	28 September 2018	Professionals delivered lectures on rabies in animals and humans, and the implementation of One Health in rabies eradication in Nigeria
Animal Hospital Unit, onaaraTODAYservices	NGO	World Rabies Day 2018	27–29 September 2018	Asking a well-to-do client to pay for one more anti-rabies vaccination of another dog. Free vaccination of dogs at Akanran village with raised funds
One Health Unibadan, University of Ibadan	Students’ association	World Rabies Day outreach	22–27 September 2019	Rabies education in some secondary schools through interactive sessions, and distribution of resource materials to the schools
NYSC scheme in collaboration with the Nigerian Veterinary Medical Association (NVMA), Ondo State Chapter	Collaboration	One Health approach to curb rabies in Nigeria	28 September 2019	A seminar on One Health approach to rabies elimination in Nigeria
Vetlane Animal Healthcare, ElSalem Estate, Lugbe, Abuja	Private Veterinary Organization	Free anti-rabies vaccinations of dogs and cats	28 September 2019	Free anti-rabies vaccines to dogs and cats at two locations in Abuja Metropolis, Nigeria
PawsConnect, Abuja	Nonprofit organization	World Rabies Day Awareness Dog Walk	28 September 2019	A 5 km Awareness Dog Walk in the city of Abuja, Nigeria. A paper presentation by a registered Veterinary Doctor from Veterinary Association, Abuja Chapter on the importance of Rabies Vaccination. Free medical attention and anti-rabies vaccination shots to dogs
IVSA Ibadan, Faculty of Veterinary Medicine, University of Ibadan	Students’ association	World Rabies Day College Symposium	28 September 2019	Sensitization of secondary school students about the importance of vaccination in rabies prevention and elimination
Carotid Animal Health Services, Egbeda, Lagos	Private veterinary practice	End rabies: collaborate, vaccinate	20–27 September 2020	A 7-day free anti-rabies vaccination in the community, alongside awareness and sensitization campaign of the general public on rabies in dogs and humans
NVMA Ogun State Chapter, Abeokuta, Ogun State	Professional body	WRD 2020	28 September 2020	Rabies Awareness Walk, Lecture, Free anti-rabies vaccination campaign, and Press Conference
The Department of Veterinary Services, Benue State Ministry of Agriculture, Nigeria Veterinary Medical Association (NVMA), Benue State Chapter and NGOs (Tesedona Foundation for Animal Health, MoboVet, War Against Rabies, Zoetis, VetConnect, Life Stock Management Services and Vaccinate 500 Project)	Collaboration	2020 WRD celebration in Makurdi, Benue State	28 September–12 October 2020	Radio Talk show, free vaccination of dogs in Makurdi metropolis and advocacy to policymakers
Animal Home LAUTECH	Students’ association	Animal Home free vaccination outreach	28 September 2020	Visit to some rural communities to vaccinate dogs for free and sensitize community members
PawsConnect, Garki, Abuja	NGO	PawsConnect WRD dog walk	26 September 2020	Dog walks, awareness on the danger of rabies to dogs and humans, and free vaccination of dogs
Isaachrist Awareness Foundation, Buari, Abuja	NGO	Rabies awareness campaign	27–28 September 2020	Door-to-door, village-to-village, city-to-city awareness program
Nigeria Veterinary Medical Association (NVMA), Kwara State Chapter	Professional body	NVMA Kwara state chapter free anti-rabies campaign	27 September–11 October 2020	A state-wide anti-rabies campaign for 5000 dogs across rural areas in the state. Flagged off by the Deputy Governor of the State
Ministry of Agriculture, Veterinary Services Department, Lagos State	State Government	Mass anti-rabies vaccination in Lagos, 2022	17 February–20 March 2021	Vaccination of about 1.5 million dogs in the state. The exercise covered sensitization, awareness campaign, demonstration of simple protocols following a dog bite, and vaccination of dogs, cats, and monkeys
Faculty of Veterinary Medicine, University of Abuja	College of Veterinary Medicine	UniAbuja World Rabies Day Symposium and vaccination campaign	28 September 2021 and 2 October 2021	Symposium on the theme; ‘Rabies: Facts, Not Fear,’ free vaccination of dogs and cats against rabies and public enlightenment in communities, market and places of worship
NVMA Enugu State Chapter	Professional body	Rabies: Facts not Fears	27–29 September 2021	Special radio program on rabies prevention and control; Rural free anti-rabies vaccination outreach; Mega anti-rabies sensitization rally; Free anti-rabies vaccination in urban regions
International Veterinary Students’ Association, Ibadan	Students’ association	World Rabies Day Schools campaign	27–28 September 2021	Visitation and enlightenment of secondary school students on rabies and dog bite prevention
University of Jos, VTH	Veterinary Teaching Hospital	UNIJOS with ARV Campaign	28 September 2021	Awareness and enlightenment of the general public on the dangers of this disease. Free anti-rabies vaccination of companion pets
Carotidvet Animal Health Services Ltd, Lagos	Private Veterinary Organization	World Rabies Day 2021	22–28 September 2021	Free rabies vaccinations at Carotidvet Animal Health Services Ltd. Online campaigns through official website and social media platforms
Nigerian Veterinary Medical Association (NVMA), Kebbi State Chapter	Professional body	WRD 2021	26–27 September 2021	Increase awareness about rabies and its control. Media outreach through the local Radio and TV stations, road rally, and free mass vaccination
Nigerian Veterinary Medical Association (NVMA), Taraba State Chapter	Professional body	WRD 2021	26–28 September 2021	Campaign, sensitization on the menace of rabies and free vaccination of dogs and cats
Nigerian Veterinary Medical Association (NVMA), Ogun State Chapter	Professional body	WRD 2021	28 September 2021	A seminar to educate the public and free anti-rabies vaccinations at designated Veterinary hospitals within the state for 1 week
Veterinary Teaching Hospital (VTH), University of Ibadan	Veterinary Teaching Hospital	Anti-rabies vaccination campaign	March 2021	A 2-day anti-rabies vaccination campaign involving free vaccination of dogs at centers strategically situated within the University community

NGO, non-governmental organization; WRD, World Rabies Day.

### Outcome of anti-rabies programs on the elimination of rabies

Using the 2022 anti-rabies program campaign hosted by Veterinary Teaching Hospital, University of Ibadan, Nigeria, as an example, the program was held for 5 days. The aim of the program is to provide free anti-rabies vaccine to dogs and provide sensitization about rabies to dog owners. Compared to other programs that had been held at VTH, the percentage of dog owners that showed to vaccinate their dog against rabies was low. Also, on the first 2 days of the 2022 program, the percentage of dog owners that showed up was higher compared to the other 3 days. Based on our experience with these available programs in Nigeria, the anti-rabies programs are successful as the incidence of rabies in human are reducing. Although some areas are still affected in which the arms of the small available programs cannot reach; that is the reason why there is a need for provision larger programs from the government that will provide the opportunity to do house-to-house sensitization, vaccination, and campaign in the rural areas.

### Challenges

While there are anti-rabies efforts done by individual or collaborative bodies in Nigeria, several obstacles prevent their efficacy. The absence of epidemiologic data in the nation is one of the limiting factors. In Nigeria, only a small percentage of dog bites are officially documented in veterinary and human facilities^11^,^12^. Additionally, only the National Veterinary Research Institute (NVRI), Vom, Nigeria, offers routine rabies virus diagnostic^13^, while some other institutions can test for research studies. The lack of integrated data on the incidence of rabies or knowledge of the major factors influencing rabies virus transmission in the nation has posed a major constraint to existing anti-rabies activities.

There is a lack of usage of the One Health approach in control of rabies in Nigeria due to limited collaborations among human doctors, veterinarians, and environmentalists. Almost 90% of the anti-rabies programs occurring in Nigeria are being controlled by veterinary practitioners and parastatals. This is a major challenge in controlling the occurrence of human rabies. Apart from individual counseling that is being done to the family of the affected rabid fellow, the human doctors offer limited anti-rabies programs to sensitize the public about rabies in Nigeria. This limits the effectiveness of anti-rabies programs. Rabies is a zoonotic disease, and for effective control of the disease, human doctors, veterinarians, and environmentalists need to put their hands together to use the One Health approach to eliminate the disease.

Another militating challenge against anti-rabies programs in Nigeria during the period is insecurities in some regions of the country. In recent years, Nigeria has experienced concern regarding the rate of crime and insecurity, including Boko Haram insurgency in Maiduguri, kidnapping, extrajudicial killing, rural killing, herder-farmer killing in the southwest, and killing of pro-Biafra protesters in the southeast^14^. All these have influenced the migration of people from less-safe regions, thus resulting in the neglect of anti-rabies movements in these regions.

Furthermore, the global pandemic coronavirus disease 2019, which effectively restricted movement worldwide, is another recent challenge for anti-rabies programs in Nigeria. Apart from the aspect of lockdown, which limits people from moving around for these programs, most attention and resources were focused on the containment of the emerging pandemic of coronavirus disease 2019^15^. This would have led to the proliferation of stray dogs, which may have played a significant role in the increasing incidence of canine rabies in Nigeria. For Nigeria to meet the 2030 goal of zero rabies, more needs to be done in terms of providing more resources for anti-rabies programs.

Moreover, due to poor knowledge about anti-rabies, there is always suboptimal participation in the programs. Only some well-informed individuals get involved in the program. This program requires a lot of people to help sensitize people, logistics, and distribution and injection of vaccines in the community.

### Recommendations

The current level of implemented programs and activities shows that rabies is still a neglected disease. National and international health officials need to understand that rabies has the highest case-fatality ratio of all human infectious diseases and, more importantly, that human death related to canine-mediated rabies could be 100% prevented by enhanced vaccine coverage in the canine population. It is germane for every veterinary unit to establish surveillance points within the state to monitor the incidence of canine rabies, which could serve as an early warning system for an outbreak of rabies. Under the approach of One Health, we implore the national health officials, the medical, vet doctors, and environmental health practitioners to collaborate in fighting against the elimination of rabies in Nigeria. Veterinarians should work in-hand with medical doctors to provide the rabies vaccination history of the animals when there is the occurrence of human rabies to ensure prompt diagnosis of the diseases. Also, veterinarians should keep an eye on the vaccination of every rabies susceptible animal they are in charge of or admitted to their clinics. All these health practitioners should be involved in anti-rabies programs to ensure the effectiveness of the program.

In the same vein, the usage of two different communication strategies is crucial for ensuring the salience of, and participation in, anti-rabies programs within a community: mobile phone text messaging and promotion by community leaders. This will increase the awareness of people with little manpower from individuals when using mobile telecommunication systems. More television and radio communication should be used for those that have access to them, and more physical mobilization should be made available for people living in the rural environment.

More funding and resources should be dedicated to anti-rabies programs to cater to stipends for people participating in the program and to draw some people’s attention to the program. Since the programs are free, the limited number of volunteers can be linked to the country’s poverty level. More people are willing to participate in what will fill their stomachs than prevent what will kill them. Also, the establishment of a comprehensive national rabies program by the Federal Government of Nigeria (FGN) will provide central coordination to these available programs. This will help in controlling the resources for the program and make the program more effective for Nigerian citizens.

Rabies is a long-time neglected tropical disease; the outbreak of new diseases should not affect the awareness of people about it so that we will not have more cases to battle after the new diseases. The Nigerian Health authorities should always make preparations for outbreaks to prevent the shock from affecting other ongoing programs to curb some old neglected diseases.

Most of the challenges associated with the eradication of rabies in Nigeria are lack of funds, resources, and infrastructure. We urge the FGN to treat the eradication of rabies before 2030 as a priority. The FGN should ensure to dedicate some part of the 2023 budget to fund a major anti-rabies program to complete the remaining part of the success of individual anti-rabies programs held in Nigeria.

## Conclusion

Anti-rabies programs in Nigeria are supported by individual and collaborative bodies. It is pertinent to hold on to these programs and create a comprehensive national program to achieve effective rabies eradication in Nigeria. Various challenges, such as lack of epidemiological data, resources, and insecurities in the country, should be addressed with effective recommendations in this write-up. As we are all fighting to eradicate rabies in 2030, we hope this piece provides us with knowledge about the great impact of the ineffectiveness of anti-rabies programs in eradicating the disease.

## Ethical approval

Not applicable.

## Patient consent

Not applicable.

## Sources of funding

None.

## Author contribution

D.E.L.-P. and R.O.A.: research conceptualization and design; R.O.A. and H.T.A.: methodology, data acquisition, and draft manuscript preparation and revision; D.E.L.-P.: supervision. All the authors read and approved the final draft before submission.

## Conflicts of interest disclosure

The authors declare that they have no conflicts of interest.

## Research registration unique identifying number (UIN)

Not applicable.

## Guarantor

Ridwan Olamilekan Adesola.

## Provenance and peer review

Not commissioned, externally peer-reviewed.
